# Effect of ethanol on innate antiviral pathways and HCV replication in human liver cells

**DOI:** 10.1186/1743-422X-2-89

**Published:** 2005-12-02

**Authors:** Courtney R Plumlee, Catherine A Lazaro, Nelson Fausto, Stephen J Polyak

**Affiliations:** 1 Department of Laboratory Medicine, University of Washington, Seattle, USA; 2 Department of Biological Sciences, Columbia University, New York, NY; 3 Department of Pathology, University of Washington, Seattle, USA; 4 Department of Pathology, University of Washington, Seattle, USA; 5 Departments of Laboratory Medicine, Microbiology and Pathobiology, University of Washington, Seattle, USA

**Keywords:** HCV, IFN, virus-host interactions, signal transduction, alcohol

## Abstract

Alcohol abuse reduces response rates to IFN therapy in patients with chronic hepatitis C. To model the molecular mechanisms behind this phenotype, we characterized the effects of ethanol on Jak-Stat and MAPK pathways in Huh7 human hepatoma cells, in HCV replicon cell lines, and in primary human hepatocytes. High physiological concentrations of acute ethanol activated the Jak-Stat and p38 MAPK pathways and inhibited HCV replication in several independent replicon cell lines. Moreover, acute ethanol induced Stat1 serine phosphorylation, which was partially mediated by the p38 MAPK pathway. In contrast, when combined with exogenously applied IFN-α, ethanol inhibited the antiviral actions of IFN against HCV replication, involving inhibition of IFN-induced Stat1 tyrosine phosphorylation. These effects of alcohol occurred independently of i) alcohol metabolism via ADH and CYP2E1, and ii) cytotoxic or cytostatic effects of ethanol. In this model system, ethanol directly perturbs the Jak-Stat pathway, and HCV replication.

Infection with Hepatitis C virus is a significant cause of morbidity and mortality throughout the world. With a propensity to progress to chronic infection, approximately 70% of patients with chronic viremia develop histological evidence of chronic liver diseases including chronic hepatitis, cirrhosis, and hepatocellular carcinoma. The situation is even more dire for patients who abuse ethanol, where the risk of developing end stage liver disease is significantly higher as compared to HCV patients who do not drink [1,2].

Recombinant interferon alpha (IFN-α) therapy produces sustained responses (ie clearance of viremia) in 8–12% of patients with chronic hepatitis C [3]. Significant improvements in response rates can be achieved with IFN plus ribavirin combination [4-6] and pegylated IFN plus ribavirin [7,8] therapies. However, over 50% of chronically infected patients still do not clear viremia. Moreover, HCV-infected patients who abuse alcohol have extremely low response rates to IFN therapy [9], but the mechanisms involved have not been clarified.

MAPKs play essential roles in regulation of differentiation, cell growth, and responses to cytokines, chemokines and stress. The core element in MAPK signaling consists of a module of 3 kinases, named MKKK, MKK, and MAPK, which sequentially phosphorylate each other [10]. Currently, four MAPK modules have been characterized in mammalian cells: Extracellular Regulated Kinases (ERK1 and 2), Stress activated/c-Jun N terminal kinase (SAPK/JNK), p38 MAP kinases, and ERK5 [11]. Interestingly, ethanol modulates MAPKs [12]. However, information on how ethanol affects MAPKs in the context of innate antiviral pathways such as the Jak-Stat pathway in human cells is extremely limited.

When IFN-α binds its receptor, two receptor associated tyrosine kinases, Tyk2 and Jak1 become activated by phosphorylation, and phosphorylate Stat1 and Stat2 on conserved tyrosine residues [13]. Stat1 and Stat2 combine with the IRF-9 protein to form the transcription factor interferon stimulated gene factor 3 (ISGF-3), which binds to the interferon stimulated response element (ISRE), and induces transcription of IFN-α-induced genes (ISG). The ISGs mediate the antiviral effects of IFN. The transcriptional activities of Stats 1, 3, 4, 5a, and 5b are also regulated by serine phosphorylation [14]. Phosphorylation of Stat1 on a conserved serine amino acid at position 727 (S727), results in maximal transcriptional activity of the ISGF-3 transcription factor complex [15]. Although cross-talk between p38 MAPK and the Jak-Stat pathway is essential for IFN-induced ISRE transcription, p38 does not participate in IFN induction of Stat1 serine phosphorylation [14,16-19]. However, cellular stress responses induced by stimuli such as ultraviolet light do induce p38 MAPK mediated Stat1 S727 phosphorylation [18].

In the current report, we postulated that alcohol and HCV proteins modulate MAPK and Jak-Stat pathways in human liver cells. To begin to address these issues, we characterized the interaction of acute ethanol on Jak-Stat and MAPK pathways in Huh7 cells, HCV replicon cells lines, and primary human hepatocytes.

## Materials and methods

### Cells and chemicals

Human hepatoma Huh7 cells were grown in DMEM containing 10% FBS, 1× penicillin, streptomycin, fungizone, 10mM L-glutamine, and 1× non-essential amino acids (all reagents were from Invitrogen; Carlsbad, CA). BB7 cells are derived from Huh7 cells and support the replication of a subgenomic HCV replicon containing a S2204I adaptive mutation in the NS5A gene [20]. FL-Neo cells are a stable Huh7 derived cell line containing a genomic length HCV replicon with the S2204I mutation in NS5A and a P1496L mutation in NS3. BB7 and FL-Neo cells were obtained from Apath, LLC. Subgenomic replicon cell lines 9–13 and 5-15-9-2-3 (referred to as 5–15 in this paper) containing different adaptive mutations [21-23] were kindly provided by Dr. Ralf Bartenschalger. Replicon cell lines were maintained in Huh7 media containing 400 μg/ml of G418 (Calbiochem; San Diego, CA). Primary human fetal hepatocytes were isolated and grown in chemically defined serum free medium as described [24]. Primary hepatocyte cultures were analyzed within 2 days of isolation. Cells were maintained in humidified incubators at 37°C with 5% CO_2_. Ethanol (AAPER; Shelbyville, KY) at concentrations of 0–200 mM, was added to cells at the same time as IFN-α (Sigma, St. Louis, MO). Relative to untreated cells, ethanol did not induce any cytotoxic or growth inhibitory effects on any of the cell types at any of the doses tested (see Additional File 1). MAPK inhibitors UO126, PD98059, and SB203508, used to inhibit p42/44, MEK1, and p38 MAPK pathways, respectively, were solubilized in DMSO and obtained from Calbiochem. ADH and CYP2E1 inhibitors 4-methylpyrazole (4-MP) and diallylsulfide (DAS) [25], were obtained from Sigma and solubilized in DMSO. In all experiments, the final concentration of DMSO was below 0.2%, so as to prevent DMSO inhibition of CYP2E1 [26].

### Transfection

The day prior to transfection, 2 × 10^5 ^cells were plated in 12-well tissue culture plates. Endotoxin free plasmid DNA was purified (Endofree kit, Qiagen; Valencia, CA), and was introduced into cells with lipofectamine 2000 according to manufacturer's recommendations (Invitrogen). Transfection efficiency was monitored by including 0.5 μg of plasmid pQ150 (provided by Dr. Jeffery Vieira), which expresses GFP under control of the constitutive EF-1α promoter. Prior to harvesting protein lysates, cells expressing GFP were visualized by fluorescence microscopy and the transfection efficiency calculated by determining the percentage of green cells to total cells. For reporter gene studies, 0.5 μg of the luciferase gene under control of the interferon stimulated response element (ISRE) in plasmid pISRE-luc (ISRE promoter; Stratagene; La Jolla, CA), was transfected into cells in duplicate or triplicate. In certain experiments a dominant negative p38 (p38 AGF) expressing plasmid [27], provided by Dr. Michael Kracht, was transfected into cells. Twenty-four hours post-transfection, ethanol, either alone or in combination with IFN was added directly to cells. Six hours later, luciferase activity was measured on cell lysates and normalized for transfection efficiency and total protein content.

### Western blot analysis

Protein lysates were quantitated by BCA Protein Assay (Pierce; Rockford, IL) and equal amounts (typically 20–30 μg) of total protein was separated on 4–20% SDS-PAGE gels. For detection of phosphorylated Stat1 proteins, Stat1 phosphotyrosine (Y701) and phosphoserine (S727) specific antibodies were used (Zymed-Invitrogen). Total Stat1 was detected using a polyclonal antibody (Zymed or Santa Cruz Biotechnology; Santa Cruz, CA). Total and phosphorylated forms of p42/44 (ERK2/1), and p38 MAPK were detected with specific antisera (Cell Signaling; Beverly, MA). Cytochrome P4502E1 (CYP2E1) was detected using polyclonal rabbit antiserum (provided by Arthur Cederbaum), while alcohol dehydrogenase (ADH) was detected with a mouse monoclonal antibody (AbCam; Cambridge, MA). HCV proteins were detected using random, de-identified HCV infected patient serum, as described [28]. Infected serum was inactivated by adding triton X-100 to 1% prior to use.

### Kinase assays

The activity of p38 MAPK in Huh7 cells was assessed via kinase assay using a kit (Cell Signaling). Briefly, cell lysates were immunoprecipitated with antibodies that recognize the phosphorylated form of p38 MAPK. After stringent washing, recombinant ATF-2 protein, a substrate for p38, was added to immunoprecipitates and incubated for 30 minutes according to manufacturer's specifications. Phosphorylated protein ATF-2 was detected by western blot.

### HCV RNA quantitation

HCV RNA was quantitated by real time RT-PCR, using a modified version of a recent procedure [29]. Total cellular RNA was isolated from replicon cells using a commercial kit (Qiagen). Ten nanograms of RNA was added to wells of a 384 well plate containing the EZ RT-PCR master mix (Perkin Elmer; Wellesley, MA). Samples were run on an ABI HT7900 real time RT-PCR machine. The RT reaction consisted of 50°C for 2 minutes followed by 60°C for 30 minutes. The PCR consisted of an initial denaturation of 2 minutes at 95°C, then 45 cycles of 95°C for 15 seconds followed by simultaneous annealing/extension at 60°C for 1 minute. For each run, dilutions of BB7 plasmid DNA (precisely quantitated using the PicoGreen DNA quantitation kit (Invitrogen)) ranging from 0–10^7 ^copies per tube were run in triplicate to generate a standard curve, which served as a reference to calculate HCV RNA copy number based on the cycle threshold (C_t_). The HCV RNA copy number is reported as copies per 10 ng total cellular RNA. Additional controls included reactions lacking template as well as RNA from Huh7 cells. For both negative controls, these samples were always negative for HCV RNA.

### ADH enzyme assay

Cells were harvested in PBS and whole cell extracts prepared via sonication. Aliquots of protein extracts were mixed with 0.1 M glycine pH 10.0 buffer, 2.4 mM β-nicotinamide adenine dinucleotide, and 33 mM ethanol, and conversion of NAD to NADH+ was monitored with a spectrophotometer at a wavelength of 340 nm. All reagents for the assay were from Sigma. As a positive control, purified human ADH (provided by Dr. Carol Stone) was also run in the assay.

### Statistics

Differences between means of luciferase readings were compared using a Student's T-test. A p-value of <0.05 was considered significant. For western blots, data were analyzed with Image J, a software version of NIH Image for the Macintosh OS × operating system. Changes in protein levels were normalized to control western blots and expressed as fold or percent change relative to controls.

## Results

### Effect of acute ethanol on Jak-Stat pathway

**Figure 1:**
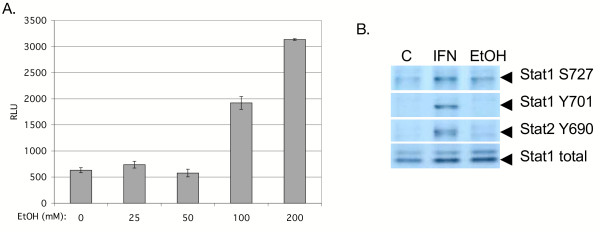
Effect of acute ethanol on the Jak-Stat pathway. A, high physiological doses of ethanol activate the ISRE. Huh7 cells were transfected with 0.7 μg of pISRE-luc, and 24 hours later, cells were stimulated with ethanol at the indicated concentrations. Protein lysates were assayed for luciferase activity 6 hours later. Error bars represent standard deviations. The experiment was repeated 6 times with similar results. B, acute ethanol induces Stat1 serine phosphorylation. Huh7 cells were left as untreated controls (C) or treated with 1,000 U/ml of IFN-α (IFN), or 100 mM ethanol (EtOH). Twenty minutes later, equal amounts of whole cell protein extracts were separated by SDS-PAGE, and blotted for phosphorylated Stat1 S727 (toppanel), Stat1 Y701 (second panel), Stat2 Y690 (third panel), and total forms of the Stat1 protein (lower panel). The figure is representative of 3 independent experiments, which yielded similar results.

Figure 1A depicts the effects of acute ethanol on the ISRE promoter in Huh7 cells. Ethanol did not appear to have significant effects on ISRE activity at 25 and 50 mM concentrations. However, at concentrations of 100 and 200 mM, ethanol caused statistically significant 3.0 (p = 0.03) and 5.0 (p < 0.001) fold increases in ISRE reporter gene activity, as compared to cells not treated with ethanol. The data suggest that high physiological doses of acute ethanol activate the ISRE, an IFN responsive promoter.

To investigate this regulation further, we analyzed levels of phosphorylated Stat1 and Stat2, which are obligatory steps for ISRE activation. Stat1 and Stat2 activation involves phosphorylation on conserved tyrosines at amino acid positions 701 and 690, respectively, while phosphorylation of Stat1 also occurs on conserved serine amino acid at position 727 and provides maximal transcriptional activation [15]. Figure 1B depicts the levels of Stat1 S727 (top panel), Stat1 Y701 (second panel), and Stat2 Y690 (third panel), and the total levels of Stat1 protein (fourth panel) in Huh7 cells. Phosphorylation of Stat1 on S727 was induced by IFN-α or 100 mM ethanol. Stat1 Y701 and Stat2 Y690 phosphorylation occurred with IFN treatment, whereas no effect was observed with 100 mM ethanol. The differences in Stat phosphorylation were not due to differences in the amount of Stat1 protein, since total Stat1 protein levels were equivalent (Figure 1B, lower panel). Similar results were also observed for primary human fetal hepatocytes (see Additional File 2) and HCV replicon cells (data not shown).

### Effect of acute ethanol on the p38 MAPK pathway

**Figure 2:**
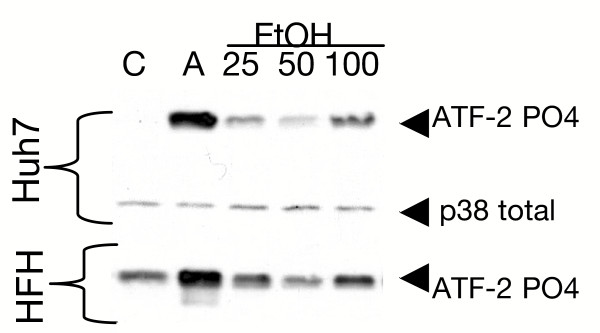
Ethanol activates p38 MAPK in human liver cell cultures. Huh7 cells or primary human fetal hepatocytes (HFH) were left as untreated controls (C) or were treated with anisomycin (A) as a positive control, or with 25, 50, or 100 mM ethanol for 30 minutes. Active p38 MAPK was immunoprecipitated from cell lysates and kinase activity measured by phosphorylation of ATF-2. The figure is representative of 2 experiments that produced similar results.

Since MAPKs are modulated by ethanol [12] and p38 MAPK is important in ISRE transcription [16,19], we next examined the effect of acute alcohol on the p38 MAPK pathway. Figure 2 depicts the effects of acute ethanol on the p38 MAPK pathway in Huh7 cells and primary human fetal hepatocytes. In these experiments, p38 kinase assays were performed. As shown in the upper panel of Figure 2, acute exposure of Huh7 cells to 25, 50, and 100 mM ethanol resulted in 61, 27, and 150-fold activation of p38 kinase activity, respectively, detected as an increase in recombinant ATF-2 phosphorylation, a natural substrate for p38 MAPK. The middle panel depicts the amounts of total p38 protein added to each immunoprecipitate. Acute ethanol at 25, 50, and 100 mM doses also activated p38 MAPK to levels 2.1, 2.2, and 5.2-fold in primary fetal human hepatocytes, relative to untreated cells (Figure 2, lower panel), although basal levels of MAPK were higher in these cells. The data suggest that acute ethanol activates p38 MAPK pathways in primary human fetal hepatocytes and Huh7 cells. Acute ethanol also activated p42/44 MAPK and SAPK in Huh7 (see Additional File 3) and BB7 replicon cells (data not shown).

### Acute alcohol stimulation of the Jak-Stat pathway involves MAPKs

**Figure 3:**
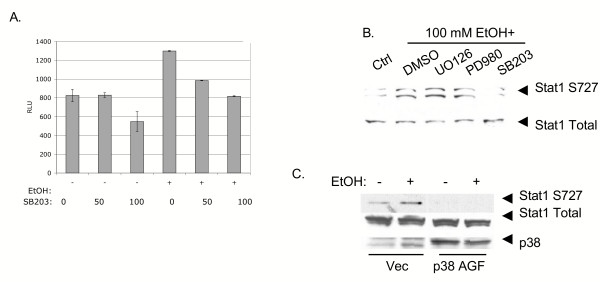
Involvement of p38 MAPK in ethanol induction of ISRE transcription and Stat1 serine phosphorylation. Panel A, Huh7 cells were transfected with 0.7 μg pISRE-luc, and 22 hours later, cells were incubated for 2 hours at the indicated μM concentrations of SB203508 (a p38 inhibitor), followed by 100 mM ethanol. Cell lysates were harvested 6 hours later and luciferase results were normalized to amounts of total cellular protein. Error bars represent standard deviations. The experiment was repeated 4 times with identical results. B, Huh7 cells were treated for 2 hours in the presence of 50 μM of various MAPK inhibitors, and stimulated with 100 mM alcohol for 20 minutes. Whole cell protein extracts were blotted for the serine phosphorylated form (S727) or total form of Stat1. The experiment was repeated twice, yielding similar results. C, Huh7 cells were transfected with control vector plasmid (Vec) or a plasmid expressing a dominant negative mutant p38 protein (p38 AGF). Twenty-four hours later, cells were not treated or treated for 20 minutes with 100 mM ethanol. Levels of S727 and total Stat1 and transfected p38 proteins were determined by western blot. The figure is representative of 2 independent experiments that produced similar results.

Since the p38 MAPK pathway cross-talks to the Jak-Stat pathway [16,19], we investigated the effect of acute alcohol on ISRE transcriptional activity and Stat1 phosphorylation in the presence of MAPK inhibitors and dominant negative mutants. We performed ISRE reporter gene experiments with IFN-α and alcohol treatments in the presence of the p38 MAPK inhibitor, SB203508. As shown in Figure 3A, SB203508 inhibited ethanol stimulation of the ISRE by up to 40%. Figure 3B presents related experiments examining the effect of small molecule inhibitors on ethanol induction of Stat1 serine phosphorylation. Huh7 cells were treated for 2 hours in the presence of DMSO carrier, UO126 (a p42/44 MAPK inhibitor), PD98059 (a MEK1 inhibitor) and SB203508 (a p38 inhibitor). Cells were then stimulated with 100 mM ethanol for 20 minutes. Ethanol induction of Stat1 serine phosphorylation was 90% inhibited by SB203508. Huh7 cells were also transfected with a vector expressing a dominant negative p38 protein (p38 AGF) [27], and the effect on ethanol induction of Stat1 serine phosphorylation was investigated. As shown in Figure 3C, expression of the p38 AGF dominant negative mutant abrogated both basal and ethanol induced Stat1 serine phosphorylation. Together, the data suggest that acute ethanol activation of p38 MAPK is partially involved in induction of ISRE transcription and Stat1 serine phosphorylation.

### Effect Of acute alcohol on HCV replicons

**Figure 4:**
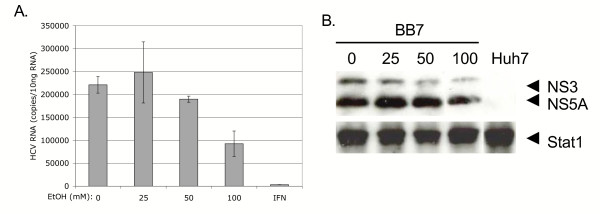
Effect of acute ethanol on HCV replication. BB7 replicon cells were treated once with 0, 25, 50, or 100 mM ethanol or 20 U/ml of IFN, and RNA and protein was harvested 72 hours later. A, HCV RNA copy number was determined by quantitative real time RT-PCR. The HCV RNA copy number is reported as copies per 10 ng total cellular RNA. Error bars represent standard deviations. B, HCV protein expression in BB7 cells treated with 0, 25, 50, and 100 mM ethanol, and control Huh7 cells. The positions of Stat1, HCV NS3 and NS5A proteins are indicated. The experiment was repeated twice with similar results.

Figure 4 depicts the effects of acute ethanol on HCV replication. BB7 cells were treated once with 0, 25, 50, or 100 mM of alcohol, or 20 U/ml of IFN-α. HCV RNA was quantitated using real time RT-PCR on equal amounts (10 ηg) of total cellular RNA isolated 72 hours after drug treatment (Figure 4A; left panel). As expected, IFN induced a significant 66-fold inhibition of HCV RNA at this time point. A single administration of 25 mM ethanol had no significant effect on HCV RNA replication, although a slight increase was noted. In contrast, 50 mM and 100 mM ethanol doses induced statistically significant inhibition of HCV RNA synthesis (p = 0.02 and p = 0.001, respectively). The doses of alcohol used did not affect BB7 cell growth, viability, or morphology (data not shown). HCV NS3 and NS5A protein expression was also inhibited in a dose-dependent fashion (1.7–5.4 fold) by ethanol (Figure 4B, right panel).

**Figure 5:**
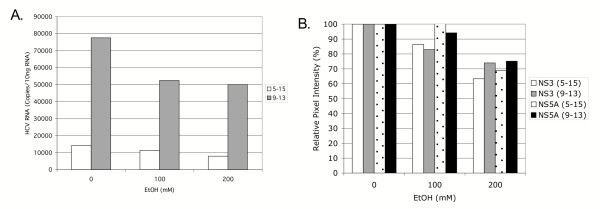
Acute ethanol inhibits the replication of other HCV replicon lines. 9–13, and 5–15-replicon cell lines were treated with 0, 100, or 200 mM of ethanol, and HCV RNA (panel A) and protein (panel B) was quantitated by real time RT-PCR and western blot analysis as described above. B., quantitation of changes in HCV NS3 and NS5A protein expression. Scanned blots were analyzed with Image J. For each lane, pixel intensities of NS3 and NS5A bands were normalized to the total Stat1 pixel intensity, and the percent change relative to untreated cells was calculated.

Since replicons acquire adaptive mutations [20,23] and it is also possible that the cells acquire genetic or epigenetic mutations during the G418 selection process and continuous culturing [30,31], we questioned whether the previous data derived from a single replicon cell line was typical of other replicons. We therefore examined the effect of acute ethanol on HCV RNA and protein synthesis in 2 additional replicon lines, 9–13 and 5–15, obtained independently from BB7 cells [21-23]. Cells were treated with 100 or 200 mM of ethanol, and HCV RNA and protein was assessed 72 hours later. As shown in Figure 5, although the basal level of HCV RNA differed considerably between 5–15 and 9–13 replicon cells, both doses of ethanol inhibited HCV RNA and protein production by up to 50%. Together the data suggest that acute ethanol inhibits the replication of several independent cell lines that support robust HCV replication. The replication of a genomic length replicon cell line, FL-Neo, was also inhibited by acute ethanol (see Additional File 4).

### Acute alcohol inhibits the IFN-α induced antiviral response towards HCV

**Figure 6:**
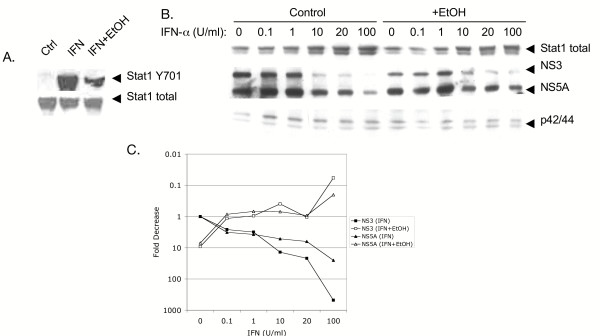
Acute alcohol inhibits the antiviral actions of IFN. A, Huh7 cells were left untreated (Ctrl), or treated with 1,000 U/ml of IFN-α alone (IFN) or with IFN-α plus 100 mM ethanol (IFN+EtOH). Proteins were probed for Stat1 Y701 (top panel), and total Stat1 proteins (lower panel). B, BB7 replicon cells were treated with or without 100 mM ethanol, followed immediately by 0, 0.1, 1, 10, 20, 100 IU/ml of IFN-α, and whole cell protein extracts were harvested 48 hours later. Equal amounts of total cellular protein were analyzed for the presence of Stat1, HCV NS5A and NS3 proteins, and p42/44 MAPK by western blot analysis. C, quantitation of HCV protein expression shown in panel B. For each lane, pixel intensities of NS3 and NS5A bands were normalized to the total p42/44 pixel intensity, and the fold decrease relative to untreated cells was calculated. The figure is representative of 2 independent experiments that produced identical results.

We examined the combined effects of alcohol and IFN-α treatment on the Jak-Stat pathway. Huh7 cells were left untreated, or treated with IFN, or IFN plus ethanol. Figure 6A demonstrates that ethanol treatment inhibited IFN-α induction of Stat1 tyrosine phosphorylation. To investigate the effect of ethanol on the IFN induced antiviral response, BB7 replicon cells were treated with or without 100 mM ethanol in the presence of varying doses of IFN-α. HCV protein levels were analyzed by western blot 48 hours later. As shown in Figure 6B, in the absence of ethanol, IFN-α inhibited HCV protein in a dose dependent fashion, and this coincided with a dose-dependent increase in total Stat1 protein, a known ISG. When cells were treated with a single dose of 100 mM ethanol, increases in HCV NS3 and NS5A proteins were detected at IFN doses of 10, 20 and 100 U/ml relative to cells treated with IFN alone. Alcohol also inhibited IFN induced up-regulation of Stat1 at these concentrations. However, at IFN concentrations of 0, 0.1 and 1 U/ml, ethanol appeared to decrease the amount of HCV NS3 and NS5A protein expression, consistent with ethanol's IFN stimulatory and anti-HCV effects presented above. Figure 6C presents a quantitative summary of the protein data based on pixel intensity, and clearly demonstrates that IFN dose-dependently inhibits NS3 and NS5A protein expression by 10–100 fold. In the absence of IFN, acute ethanol inhibits NS3 and NS5A protein expression by 10-fold. However, acute ethanol prevents IFN-α-mediated clearance of HCV proteins. Similar effects were observed for HCV RNA production (data not shown). The data indicate that ethanol inhibits the antiviral actions of exogenously added IFN.

### Expression of alcohol metabolizing enzymes in human liver cell cultures

**Figure 7:**
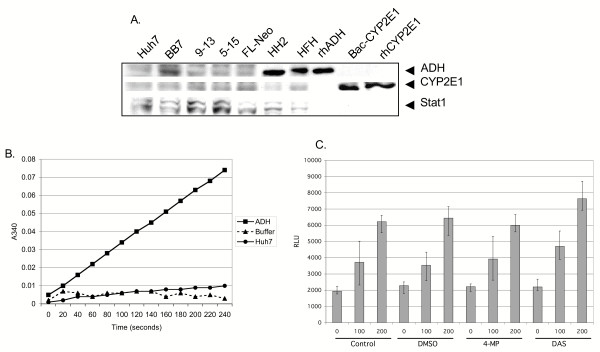
Characterization of ethanol metabolizing enzymes in human liver cell cultures. A, western blot analysis of ADH1 and CYP2E1 expression levels in Huh7, BB7, 9–13, 5–15, and FL-Neo cells. Positive controls for ADH included primary human fetal hepatocytes (HFH) [24], and a well differentiated immortalized human liver cell line, HH2 (developed in NF's lab), while controls for CYP2E1 expression included baculovirus expressed CYP2E1 and purified CYP2E1. Western blots were probed with a monoclonal antibody against human ADH, and polyclonal rabbit antiserum against CYP2E1 and Stat1. B, ADH enzyme activity. Huh7 cells were harvested in PBS and whole cell protein extracts prepared via sonication. Conversion of NAD to NADH+ was monitored at a wavelength of 340 nm as described in the Materials and Methods. Purified ADH served as a positive control for ADH activity. C, effect of CYP2E1 and ADH inhibition on ethanol activation of the ISRE. Huh7 cells in 96 well plates were transfected in triplicate with 50 ηg of ISRE-luc and 12 hours later, were treated with 5 mM of the ADH inhibitor 4-MP and 10 mM of the CYP2E1 inhibitor DAS for an additional 12 hours. Cells were also separately exposed to 0.1% DMSO, as an additional control for possible solvent effects. Cells were then treated with 0, 100, or 200 mM ethanol, before luciferase activity was measured by BriteLite assay. Error bars represent standard deviations. The experiments were repeated twice with identical results.

Since ethanol can exert differential effects on cells depending on whether it is metabolized or not [32], the expression and activity of ADH and CYP2E1, the major ethanol-metabolizing enzymes, was examined in Huh7 and replicon cells. Figure 7A depicts western blot analysis of ADH (top panel), CYP2E1 (middle panel), and Stat1 (lower panel) protein expression in Huh7, BB7, 9–13, 5–15, and FL-Neo cells. Immortalized human hepatocytes (HH2), primary human fetal hepatocytes (HFH), and purified human ADH served as positive controls for ADH, while lysate from cells that were infected with a baculovirus expressing human CYP2E1 [33], as well as purified CYP2E1, served as controls for CYP2E1. All replicon and Huh7 cultures expressed very low to undetectable levels of ADH and CYP2E1 protein. To determine if Huh7 cells expressed a functional ADH enzyme, ADH enzyme assays were performed using purified human ADH as a positive control. Figure 7B demonstrates that purified ADH showed a linear increase in absorbance over time, while buffer alone remained at background levels. In contrast, Huh7 cells expressed minimal ADH enzymatic activity, with only slight increases over background detected after 3 minutes. Figure 7C demonstrates that ethanol induction of ISRE transcription was not affected in the presence of the ADH and CYP2E1 inhibitors, 4-MP and DAS. Note that the concentrations of 4-MP (5 mM) and DAS (10 μM) used in this assay were derived from a previous study [25]. At these concentrations, 4-MP and DAS had no effects on cell viability or proliferation (data not shown). The data indicate that Huh7 and replicon cells express low to undetectable levels of ADH and CYP2E1 proteins, and further suggest that the effects of ethanol on innate antiviral pathways is not due to ethanol metabolism via ADH or CYP2E1 in this model system.

## Discussion

In the current study, it was demonstrated that high physiological doses of acute ethanol induces Stat1 serine phosphorylation and ISRE transcription. Given alone, ethanol appears to inhibit HCV replication in several independent replicon cell lines, and this is in part mediated by a Jak-Stat transduced antiviral response. In contrast, in the presence of exogenously added IFN-α, ethanol partially inhibits the antiviral actions of IFN-α, involving inhibition of IFN-α induced Stat1 tyrosine phosphorylation. Analysis of the effects of chronic ethanol administration on basal and IFN-α induced signaling responses is currently in progress.

**Figure 8:**
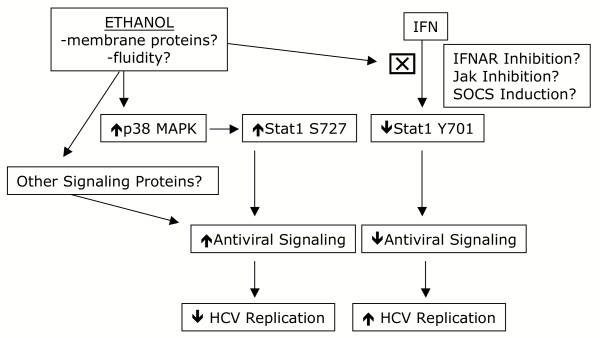
Summary of effects of acute ethanol on HCV replication. Ethanol effects in this system are independent of ethanol metabolism and as such may involve ethanol-induced perturbations in cell membranes, such as membrane fluidity. Left side, acute ethanol activates p38 MAPK which leads to Stat1 serine phosphorylation, Jak-Stat signaling and inhibition of HCV replication. Activated Stat1 may be involved in ISRE transcription but it is possible that other ISRE binding transcription factors such as Stat3 are involved in this process. Right side, ethanol inhibits the antiviral actions of exogenously applied IFN and this involves inhibition of IFN-induced Stat1 tyrosine phosphorylation, decreased Jak-Stat signaling and increased HCV replication in the presence of IFN. Inhibition of Jak-Stat signaling may involve ethanol perturbation of IFN-α induced changes in membrane fluidity, inhibition of IFN binding to its receptor, direct inhibition of Jak kinases, and/or induction of negative regulators of the Jak-Statpathway such as SOCS proteins.

We also found that acute exposure of human liver cells to physiological doses of ethanol activates the IFN system via the MAPK pathway. The data suggest that ethanol induces cross talk between the p38 MAPK and Jak-Stat pathway (Figure 8). Additional evidence for cross talk between these pathways derives from a study indicating that ERK2 binds to the α-chain of the IFN α/β receptor and STAT1 [34], and JAK2 may be required for MAP kinase pathway activation [35]. Furthermore, HCV proteins such as NS5A interact with and modulate MAPK and related pathways such as Grb2, Ras-ERK, and phosphoinositol 3 kinase (PI3K) [36-40]. However, p38 kinase activity, which is important in IFN-α and IFN-γ induced transcription, is not involved in IFN induced Stat1 serine phosphorylation [16,19]. Thus, induction of Stat1 serine phosphorylation by ethanol described in the current report may be mechanistically similar to UV-stress induced activation of Stat1 by p38 MAPK [18].

Recent studies have demonstrated that alcohol abuse may be associated with increased HCV RNA titers in patients [9]. This could be due to an increase in release of HCV RNA from alcohol-damaged hepatocytes, a direct stimulatory effect of alcohol on HCV replication, or modulation of innate and acquired immune responses to HCV. A single published report by Zhang and colleagues found that ethanol stimulates HCV replication in the replicon system [41], while our data indicate that acute ethanol inhibits HCV replication. There are several explanations for the divergent results. First, different stable replicon cell lines were used in our study as compared to the published study, so it is very likely that both the replicons [21] and Huh7 cells [30,31] are genetically different. Second, in Zhang's study, alcohol was added to replicon cells daily, so 48 and 72-hour time points actually received 2 and 3 daily doses of ethanol. This is in direct contrast to our experimental design where a single "shot" of alcohol was given. Nonetheless, chronic ethanol treatment of cells for 3 consecutive days further inhibited HCV replication in our system (data not shown). Third, in our study, the observed effects on the IFN system and HCV replication appeared to be due to the direct action of ethanol, rather than via ethanol metabolism, as reported in the Zhang study [41]. However, the dose of the ADH inhibitor 4-methypyrazole used in Zhang's study was 0.1 μM, 50,000 fold lower than the 5 mM dose used in our study, and the dose used in a seminal study demonstrating the effect of various inhibitors of alcohol metabolism [25]. Further evidence for a direct effect of ethanol for the observed results in our study stems from the observation that all replicon and Huh7 cells expressed low to undetectable levels of ADH and CYP2E1 protein, and ethanol still induced ISRE transcription in the presence of ADH and CYP2E1 inhibitors. The Zhang study did not measure ADH and CYP2E1 protein expression. Furthermore, in our studies, the effects of ethanol on the Jak-Stat pathway occurred at an ethanol concentration of 100 mM, well above that of the K_m _for ADH (1–5 mM) and CYP2E1 (16 mM) [42]. Moreover, high-dose ethanol has been previously shown to activate IFN-β-dependent antiviral activities [43], reminiscent of the data reported in the present study. Collectively, our data suggest that ethanol acts directly on cells to modulate hepatocyte signaling pathways.

Exactly how ethanol induces these signaling responses is currently under investigation. Ethanol is known to act on lipids in cell membranes as well as interact directly with membrane proteins [44-47], so it is possible that changes in membrane fluidity (defined as the physical state of the phospholipids in terms of rate and angular motion) induce downstream signal transduction events (Figure 8). In terms of the activation of the Jak-Stat pathway by acute ethanol, it is possible that besides Stat1, other proteins with the capacity to bind ISRE-like sequences are involved in ethanol induced ISRE transcription. A possible candidate is Stat3, since Stat3 is modulated by ethanol [48]. Indeed, preliminary data suggest that Stat3 is also modulated by acute ethanol in our system (data not shown). As for ethanol inhibition of IFN-induced Stat1 tyrosine phosphorylation and antiviral actions, several mechanisms might be operative (Figure 8). Since IFN-β has been shown to modulate plasma membrane fluidity [49], ethanol might inhibit IFN-α induced changes in membrane fluidity. Other possible mechanisms include ethanol inhibition of IFN-receptor interactions, or induction of negative regulators of the Jak-Stat pathway such as suppressors of cytokine signaling (SOCS) proteins. For example, SOCS-1 inhibits IFN signaling by binding Jaks to prevent Stat phosphorylation [50]. Also of note is the observation that ethanol doses of 1–20 mM did not affect HCV replication (Figure 4A), so it is possible that ethanol-induced blockade of IFN antiviral activity is more relevant in vivo. The data presented herein highlight the complexity, and emphasize the need for further study of the cellular response to acute and chronic alcohol, on innate antiviral signaling pathways and HCV replication.

In conclusion, acute ethanol treatment of Huh7 hepatoma, HCV subgenomic and genomic-length replicon cells, and primary human fetal hepatocytes has multiple effects on innate cellular defense pathways. In particular, high physiological doses of ethanol can activate antiviral responses and inhibit HCV replication, whereas it can also inhibit the IFN-α induced antiviral response against HCV replication. The data suggest that the effects of alcohol on the IFN system are not simply a stimulation or inhibition, but rather reflect highly complex processes involving cross-talk of a number of signaling pathways. The net effect of ethanol likely depends on whether ethanol is given acutely or chronically, the dose of ethanol, and whether alcohol is metabolized or not.

## Abbreviations

ADH: alcohol dehydrogenase

CYP2E1: cytochrome P450 2E1

DAS: diallysulfide

ERK: extracellular regulated kinase

HCV: hepatitis C virus

IFN: interferon

IFN-α: interferon alpha

ISG: interferon-stimulated gene

ISGF-3: interferon stimulated gene factor 3

ISRE: interferon stimulated response element

Jak: janus associated kinase

MAPK: mitogen activated protein kinase

RLU: relative light units

Stat: signal transducer and activator of transcription

4-MP: 4-methypyrazole

## Supplementary Material

Additional File 1 Effect of acute ethanol on Huh7 (panel A) FL-Neo (panel B) genomic length replicon cell viability and proliferation. Cells were treated with once 0, 50, or 100 mM ethanol, and incubated at 37°C humidified incubator with 5% CO_2 _for 72 hours. Cells were lysed and total cellular ATP content measured by luciferase assay using the ATPlite system (Perkin Elmer). Error bars represent standard deviations of quadruplicate cultures. The experiment was repeated three times with identical results. Click here for file

Additional File 2 Ethanol activation of Stat1 serine phosphorylation in primary human fetal hepatocytes. Cells were treated with 0, 25, and 50 mM ethanol or separately with 100 U/ml of IFN-α for 30 minutes, and blots were probed for Stat1 S727, Y701 and total proteins. The experiment was repeated twice with identical results. Click here for file

Additional File 3 Ethanol activates p42/44 MAPK in Huh7 cells. Huh7 cells were grown in 0.5% serum-containing media for 48 hours, and stimulated with ethanol alone at the indicated concentrations, or with ethanol and 20% serum-containing medium. Thirty minutes later, equal amounts of whole cell protein extracts were separated by SDS-PAGE and blotted for phosphorylated forms of p42/44 (panel 1) or JNK (panels 2), or total forms of p42/44 (panel 3). Click here for file

Additional File 4 Acute ethanol inhibits HCV replication in a genomic length replicon cell line. FL-Neo replicon cells were treated with 0, 100, or 200 mM of ethanol, and HCV RNA was quantitated by real time RT-PCR. The HCV RNA copy number is reported as copies per 10 ng total cellular RNA. Error bars represent standard deviations. Click here for file
