# Supplementary figures and images for: Effect of ethanol on innate antiviral pathways and HCV replication in human liver cells
Source: Virol J. 2005 Dec 2;2:89. doi: 10.1186/1743-422X-2-89 (PMC1318489; doi:10.1186/1743-422X-2-89)

**A. Huh7**

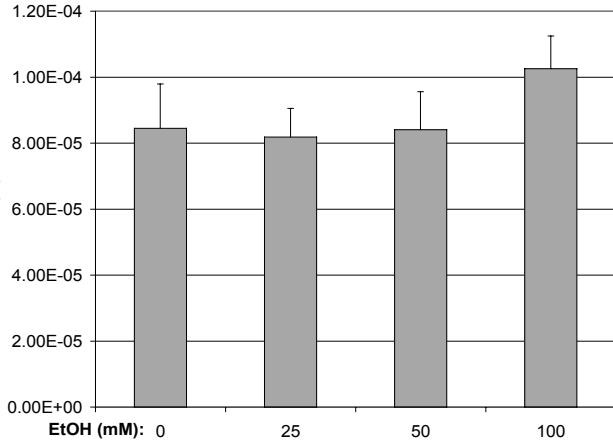

**B. FL-Neo**

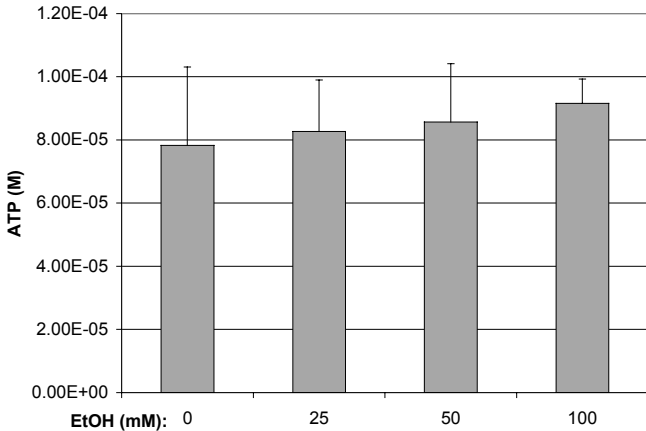

Supplement: Additional File 1 — Effect of acute ethanol on Huh7 (panel A) FL-Neo (panel B) genomic length replicon cell viability and proliferation. Cells were treated with once 0, 50, or 100 mM ethanol, and incubated at 37°C humidified incubator with 5% CO2 for 72 hours. Cells were lysed and total cellular ATP content measured by luciferase assay using the ATPlite system (Perkin Elmer). Error bars represent standard deviations of quadruplicate cultures. The experiment was repeated three times with identical results. [file 1743-422X-2-89-S1.pdf]

EtOH (mM)

0

25

50

IFN- $\alpha$

Stat 1 S727

Stat 1 Y701

Stat1 total

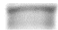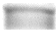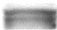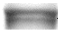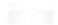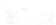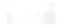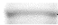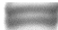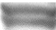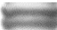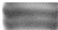

Supplement: Additional File 2 — Ethanol activation of Stat1 serine phosphorylation in primary human fetal hepatocytes. Cells were treated with 0, 25, and 50 mM ethanol or separately with 100 U/ml of IFN-α for 30 minutes, and blots were probed for Stat1 S727, Y701 and total proteins. The experiment was repeated twice with identical results. [file 1743-422X-2-89-S2.pdf]

Huh7

|        |   |   |    |    |     |    |    |     |
|--------|---|---|----|----|-----|----|----|-----|
| EtOH:  | - | - | 25 | 50 | 100 | 25 | 50 | 100 |
| Serum: | - | + | -  | -  | -   | +  | +  | +   |

1

2

3

p44 PO4  
p42 PO4

SAPK/JNK PO4

p44 total  
p42 total

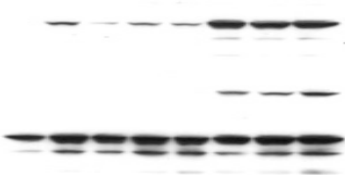

Supplement: Additional File 3 — Ethanol activates p42/44 MAPK in Huh7 cells. Huh7 cells were grown in 0.5% serum-containing media for 48 hours, and stimulated with ethanol alone at the indicated concentrations, or with ethanol and 20% serum-containing medium. Thirty minutes later, equal amounts of whole cell protein extracts were separated by SDS-PAGE and blotted for phosphorylated forms of p42/44 (panel 1) or JNK (panels 2), or total forms of p42/44 (panel 3). [file 1743-422X-2-89-S3.pdf]

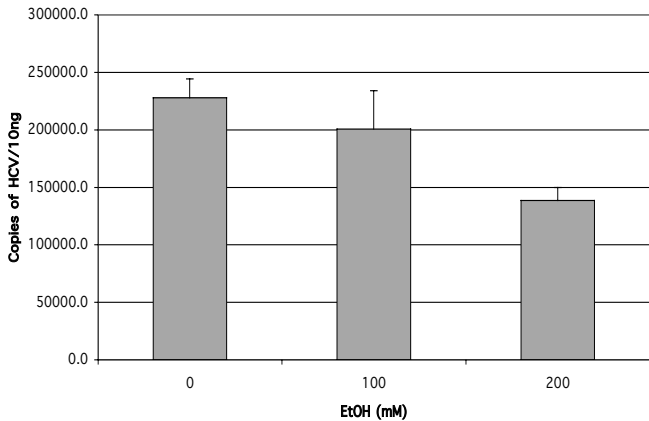

Supplement: Additional File 4 — Acute ethanol inhibits HCV replication in a genomic length replicon cell line. FL-Neo replicon cells were treated with 0, 100, or 200 mM of ethanol, and HCV RNA was quantitated by real time RT-PCR. The HCV RNA copy number is reported as copies per 10 ng total cellular RNA. Error bars represent standard deviations. [file 1743-422X-2-89-S4.pdf]
